# Testing Mortars for 3D Printing: Correlation with Rheological Behavior

**DOI:** 10.3390/ma17205002

**Published:** 2024-10-12

**Authors:** Ta Minh Phuong Bao, Muy Yeakleang, Sandra Abdelouhab, Luc Courard

**Affiliations:** 1 Urban and Environmental Engineering, Building Materials, University of Liège, 4000 Liège, Belgium; yeakleang.muy@uliege.be; 2 Belgian Ceramic Research Center, 7000 Mons, Belgium; s.abdelouhab@bcrc.be

**Keywords:** 3D printing concrete, mortars, shear level, shear stress, viscosity, consistency, rheology measurements

## Abstract

Three-dimensionally printed concrete is a transformative technology that addresses housing shortages due to population growth and enables innovative architectural designs. The objective of this study is to investigate the connection between a conventional test and the rheological properties of 3D-printed concrete. A more precise assessment of material quality based on traditional evaluation techniques is proposed. Standard tests are conducted to evaluate the consistency of 3D-printed concrete materials. Complementarily, a rheometer is employed to accurately measure key rheological properties, thereby establishing a link with empiric testing methodologies. The correlation between the flow table test and rheological coefficients, such as yield stress and viscosity, has been identified as the most effective in basic experiments for evaluating material behavior. This approach allows for a preliminary assessment of printability without the need for additional complex equipment. The study has successfully established a relationship between flow table tests and rheological parameters. However, further research involving a broader range of materials and print-test experiments is essential to enhance the correlation between other conventional testing methods and rheometer results.

## 1. Introduction

The introduction of 3D printing technology into the construction industry represents a revolutionary shift, allowing for the creation of intricate designs and complex structures with exceptional precision and efficiency [1,2,3,4]. This technology, poised to become a significant trend in residential buildings, offers several notable advantages [5]. Traditional construction methods utilizing formwork have seen little change since their inception in the Roman Empire [6]. However, contemporary challenges, such as housing shortages driven by population growth, urbanization, and economic factors, necessitate innovative construction approaches. Three-dimensional printing addresses these issues effectively, enabling the rapid construction of houses, up to 20 times faster than conventional methods, while enhancing component durability and removing architectural design constraints [7,8]. Consequently, 3D printing holds the potential to redefine the future of construction.

Three-dimensionally printed concrete is a specially formulated mixture designed to be pumped and extruded through a printing system. The success of this technology requires precise control over the material’s stability throughout the construction process. This can be evaluated through several tests, with one of the most used being the flow table test. Y. Zhang et al. [9] highlighted that in the context of 3D-printed concrete, the key parameter corresponding to this experiment is flowability. This is assessed by measuring the spread diameter of the concrete mixture at intervals of 0, 15, 30, 45, and 60 min after mixing. The study demonstrates that the concrete mixtures for 3D printing exhibit excellent shape retention, with only a 4–6 cm reduction in flow after 60 min, starting from an initial flowability of 26–30 cm. Another study conducted by A.R. Arunothayan et al. [10] utilized the flow table test to evaluate the workability of 3D-printed mortar. The spread diameter of the material ranged from 12.4 to 15.1 cm (with the original diameter being 10 cm) after 25 table drops. The relatively low flowability is attributed to the high content of metal fibers used in each mix, which is characteristic of ultra-high-performance concrete for 3D printing. Y.W.D. Tay et al. [11]’s study introduced a unique approach to evaluating the printability of 3D concrete printing. They used the flow table test in their experiments to assess the printability region of the mixtures, focusing on two key parameters: the slump flow test and the slump test. Through a series of simple experiments, the researchers evaluated the printability of the 3D mixture by assessing parameters such as the pumpability index, surface quality, and maximum printed height. Based on these evaluations, they successfully established a printable region for the material.

Additionally, there are other methods for assessing the uniformity of 3D-printed concrete. Some studies utilize the V-funnel test to evaluate the printability of fresh 3D-printed concrete, while others combine it with the pistol test. The V-funnel test, employed in [12] trials by Pham et al., confirmed its perfect alignment with the experimental results in evaluating a new 3D-printed concrete mix. The pistol test is a smaller-scale extrusion system like an actual 3D printer, but it is very simple [13]. The advantage of this method is that it provides a preliminary evaluation of the buildability of the mixtures without the need for large-scale printing, which can be wasteful. Another interesting method to assess the stability of a material is by measuring the penetration of a cone tip into the specimen (fall cone test), which evaluates the material’s resistance to the free fall of the cone. A study by Varela et al. [14] demonstrated that this test could be used to assess the thixotropy of a material, while research by A. U. Rehman [15] et al. revealed that the fall cone penetrometer significantly underestimated the structural buildup rate for stiff mixtures and was less effective for accelerated mixtures; in contrast, the fast penetration tests overestimated yield stress values compared to other instruments.

Tests like the flow table test alone are insufficient for controlling material quality. More precise measurements of fluid behavior are needed to identify the key characteristics for quality control. Therefore, studying the rheological properties of 3D-printed concrete is essential. Research on the rheology of 3D-printed concrete focuses on the fresh state of materials, with Le et al. [16] identifying three key properties: extrudability, workability, and open time. This study is among the first to explore the fresh state of 3D-printed concrete and how to design a “printable” mixture, though no specific rheological parameters were provided. Subsequent studies offered more detailed insights into the rheology and properties of 3D-printed concrete, introducing viscosity and yield stress as key parameters for easily evaluating material quality [15,16]. The yield stress and viscosity [17,18,19,20] are directly related to the pumpability, extrudability, and buildability [21,22] of 3D-printed concrete, which are three critical concepts for the advancement of this technology.

Pumpability refers to the ease with which the mortar can be mixed, pumped, and placed. Pumpability can be affected by yield stresses. Previous studies [23,24] have demonstrated that, in conventional concrete, the material behaves as a plug when flowing through a pipe. The coarse aggregate migrates toward the center, creating a plug, while a deformable lubricating layer develops along the inner wall of the pipe. A material with high yield stress may easily clog during pumping. Consequently, the material used for 3D-printed concrete must possess a low yield stress, as demonstrated by research conducted by Luiza R. M. de Miranda [25]. The yield stress values within the pumpable zone of the mixes range around 60 Pa. Extrudability expresses the ability of mortar to be pushed through the nozzle of a 3D printer without clogging and to form a smooth, continuous filament. According to Atta Ur Rehman and Jung-Hoon Kim [26], extrudability can be affected by mix proportion, 3D printer parameters (pumping system, pipe length, etc.), and rheological properties. L. Yang et al [27]. agreed with the findings of Atta Ur Rehman et al. [26] concerning the impact of the print nozzle on the extrudability of the material. They identified several key factors, including nozzle shape, interlayer height, and printing speed, that significantly affect the extrudability of concrete mixtures. Le et al. [16] conducted significant research on the composition of 3D-printed concrete mixtures. Their findings indicated that mixtures with a high sand ratio are more prone to causing pump blockages during the printing process. Additionally, they determined that mixtures with a yield stress in the range of 0.3 to 0.9 kPa are optimal for enhancing extrudability. Another study [28] corroborated the findings of Rehman et al. regarding the impact of mixture proportions on the extrudability of printed concrete. The research indicates that the maximum allowable values for the glass-to-binder ratio, fineness modulus, and nano-clay content in 3D concrete are 0.2%, 0.9, and 2.4, respectively. Exceeding these thresholds renders the material un-extrudable within the pumping system. Buildability is the capacity of the mortar to retain its shape and support subsequent layers without collapsing. The study of S. Muthukrishnan et al. [29] identified several factors closely associated with buildability, as well as various methods to enhance the buildability of 3D-printed composites. Two notable approaches are: accelerating the development of yield strength by incorporating specialized admixtures and increasing the rate of setting. Both methods aim to raise yield stress and viscosity after the extrusion process is complete, thereby improving the material’s buildability. A separate investigation [30] was conducted with the objective of identifying tests to assess the buildability of the material. The findings indicated that a yield stress between 500 and 600 Pa, along with a viscosity of approximately 8 Pa·s, would result in a favorable overlayer, and it was concluded that buildability is strongly influenced by the build-up of cement-based materials, as well as the printing thickness and the time interval between prints. Several parallel studies [31,32,33,34,35,36] have documented that extending the interlayer interval time could provide sufficient time for early strength development in the lower layers of 3D-printed concrete structures, thereby enhancing the buildability of the material.

Therefore, to accurately evaluate the fresh properties of 3D-printed concrete, in addition to basic tests such as the flow table test, V-funnel test, and pistol test, it is essential to perform precise rheological measurements for viscosity and yield stress. This is necessary to thoroughly understand the three key properties: extrudability, pumpability, and buildability. Three-dimensionally printed concrete is a unique material requiring contrasting rheological properties, making it challenging to address the values of yield stress and viscosity. In its fresh state, while moving through the pumping system, low values of both are needed. However, to enhance buildability, as previously discussed, high viscosity and a sufficiently large yield stress are necessary. In addition, having high thixotropy is also a key characteristic associated with buildability [11,34,37,38]. Achieving the right balance among these factors is essential for the success of this technology [39,40]. In the study conducted by Moeini et al. [41], the yield stress value was found to be in the range of 0.1 kPa, with a plastic viscosity of 1.9 Pa·s. Similarly, Zhang et al. [42] and Nair et al. [43] reported yield stress values ranging from 0.1 to 0.3 kPa; however, their viscosity measurements were notably higher, at approximately 3.5 to 4.1 Pa·s in Zhang et al.’s study and around 4.2 Pa·s in Nair et al.’s research. In contrast, the study by Jayathilakage et al. [44] presented yield stress and viscosity values for 3D-printed concrete of up to 1.2 to 1.8 kPa and 24.2 to 47.1 Pa·s, respectively. This study is noteworthy because Rehman et al. [26] emphasized that the yield stress of 3D-printed concrete typically falls within the range of 0.1 to 0.5 kPa, with viscosity values around 2.0 to 4.0 Pa·s. The variation in these measurements is largely influenced by factors such as printer structure, printing nozzle, and pump pressure, making it unsurprising that significant differences arise in the results obtained across different studies.

Research conducted worldwide has demonstrated methods for assessing material consistency through practical experiments (flow table test, V-funnel test, etc.), successfully evaluating the properties of 3D-printed concrete based on rheological criteria such as viscosity, yield stress, and thixotropy. However, few studies have thoroughly explored the correlation between these traditional tests and the results obtained from rheometers. Establishing this link could provide a direct connection between classical experiments, like the flow table test, and rheological values such as viscosity and yield stress. This would not only save time, but also facilitate more accurate assessments of material behavior using simple, traditional testing methods. 

In addition to identifying the most suitable test for evaluating the behavior of 3D-printed concrete (mortar), this study aims to establish a relationship between basic concrete tests and rheological parameters. The tests involved include the flow table test, pistol test, V-funnel test, and fall cone test. To measure rheological values, the RheoCAD rheometer will be used. This rheometer, specifically designed for cementitious materials, features a four-blade system and operates using the same principles and measurement methods as the ICAR viscometer [45].

## 2. Materials and Methods

### 2.1. Three-Dimensional Printing Mortar 

Four different mixes were used. The first mixture was a reference mortar prepared in accordance with standard EN:206 [46], consisting of 1350 g of standard sand, 445 g of cement, and 225 g of water; this is, of course, not a 3D printing mortar, but it was used as a well-known reference. The next two mixes (named the A and B mixtures, respectively) were commercial products designed for 3D-printing processes. 

Mixture A was selected for its specific rheological properties compared to a conventional mortar. It demonstrates excellent pumpability and extrudability through the printer, as well as strong flexural and compressive strength. Additionally, it offers enhanced resistance to seawater and sulfate environments. Importantly, Mixture A features compensated shrinkage, which prevents 3D-printed components from cracking after curing—a critical factor in the printing process. 

Mixture B also offers high compressive strength and possesses several advantages for the 3D-printing process. These include high thixotropic properties, which enable users to customize and create intricate architectural designs, as well as its ability to be stacked in multiple layers. Additionally, its short setting time allows for the rapid relocation of components to the desired location, significantly saving time.

Given the objective of comparing the rheological behavior of materials, researching existing 3D-printed mortars is useful for establishing reference characteristics. Details about these commercial products are shown in Table 1. 

A newly developed mix, Mixture C, was manufactured for the project. The primary goal of Mixture C is to produce refractory concrete while maintaining key properties essential for 3D printing, such as pumpability, extrudability, and buildability. Due to its high-temperature resistance, Mixture C consists of three main components: 73 wt.% tabular alumina, 10 wt.% reactive alumina, and 5 wt.% calcium aluminate cement, along with minor components like 1.5 wt.% CaO and 0.1 wt.% of a stiffness agent.

The original water mixing contents (recommended by the manufacturers) for mixtures A, B, and C are 10 wt.%, 18 wt.%, and 8 wt.%, respectively. To evaluate the robustness of the mixtures to the 3D printing process, the water content for each, except the reference mixture, was adjusted to be either one or two percent higher or lower than the original. This resulted in 10 different mixtures being tested (Table 2).

### 2.2. Testing Methods for Consistency 

Several tests exist for measuring consistency of mortars, including self-compacting mortars, but not for 3D-printed mortar. Before developing a new procedure, it is of prime importance to check whether classical tests are suitable for discriminating the rheological performances of 3D-printed mortars. 

**Figure 1:**
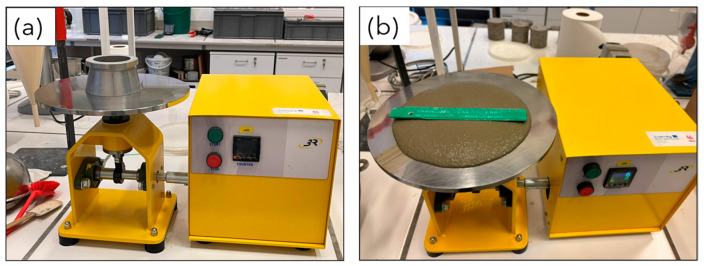
(**a**) The flow table test; (**b**) sample on the flow table after raising 15 times.

The first test considered is the flow table test (Figure 1a), which is performed according to the standard EN 13395-1:2002 [47]. This test allows the spread of the mortar to be measured to determine its workability. After raising the table vertically 15 times, the spreading diameter is measured (Figure 1b). This test is conducted four times at intervals of 0, 10, 20, and 30 min after mixing, respectively, to assess the time-dependent workability of the mortar.

**Figure 2:**
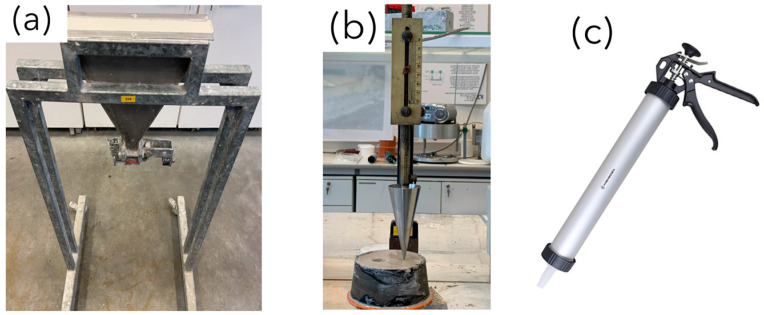
(**a**) V-funnel test, (**b**) fall-cone test, (**c**) pistol test.

The second test proposed is the V-funnel test (Figure 2a), conducted according to the standard EN 12350-9:2010 [48]. This test measures the time required for the mortar to flow out of a funnel. Although primarily designed for self-compacting mortar (SCM), the similar initial behavior of 3D-printed mortar justifies its use for evaluating consistency. 

The fall-cone test (Figure 2b), which follows the standard BS 1377-2:1990 [49], is another method used for assessing the consistency of 3D-printed mortar. This test, originally designed to determine the liquid limit of soil, measures the penetration depth of a cone into the sample. The cone is positioned on the surface of the test specimen and then dropped freely into the mortar as illustrated in the Figure 2b. By observing how deeply the cone penetrates the material, valuable insight into the mortar’s resistance to deformation and its overall consistency can be deduced. 

Finally, the pistol test (Figure 2c) is a widely used method in 3D printing construction to evaluate the buildability and extrudability of the mortar. The pistol has a 1.6 cm diameter nozzle, a body length of 40 cm, and a body diameter of 5.1 cm. This test involves printing a small component consisting of five layers to observe the material’s behavior during the printing process. This test is crucial for understanding how the mortar could perform in practical applications, as it simulates on-site conditions and assesses the material’s ability to extrude smoothly and build up layer by layer without collapsing.

### 2.3. Rheological Parameters Measurement

Concrete and mortar are non-Newtonian fluids; the most common model to illustrate their behavior is the Bingham model defined by Equation (1):

(1) $$\tau =\tau_{0}+\mu \dot{\gamma }$$

where τ and $\tau_{0}$ represent the shear stress and yield stress, respectively; μ is the plastic viscosity; and $\dot{\gamma }$ is the shear rate.

The two most critical parameters are yield stress (τ_0_) and viscosity (μ). Materials intended for 3D-printing technology must meet several specific requirements. They must be fluid enough to be transported through the pipe system and subsequently printed, yet robust enough to support multiple layers without collapsing. The suitability of a material for 3D printing is judged based on criteria such as pumpability, extrudability, and buildability. Pumpability and extrudability are related to yield stress, while buildability is influenced by both yield stress and viscosity [50]. 

**Figure 3:**
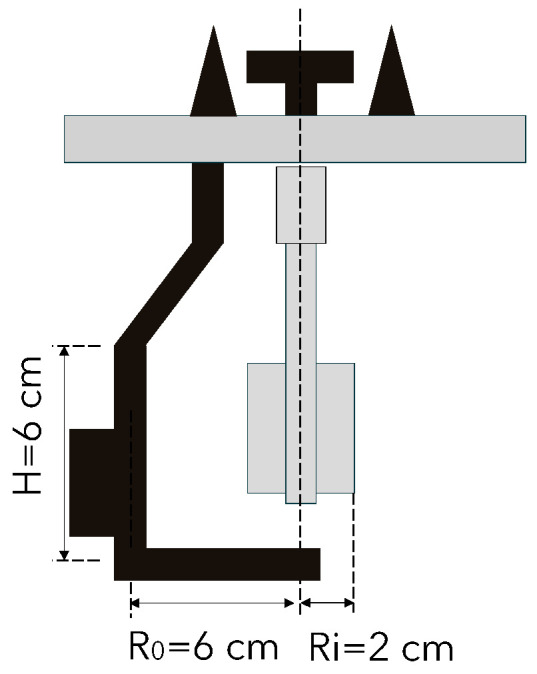
Principle and geometry of the blade of RheoCAD.

The rheological characteristics are measured using a rheometer, model RheoCAD (CAD Instrument, Les Essarts-le-Roi, France). This device is specifically designed to measure the viscosity and yield stress of mortar. The RheoCAD features a four-blade rotation mechanism with a maximum capacity of 3 L of mortar. The blade dimensions are shown in Figure 3.

The RheoCAD records values in terms of rotation speed (N—rounds per minute) and torque (T—N·cm) based on the equation T = G + H × N. This equation is derived from the Reiner equation, Equation (2), where the parameters G and H are used to calculate the yield stress and viscosity of the material:

(2) $$\tau_{0}=\frac{\left(\frac{1}{R_{i}^{2}}-\frac{1}{R_{o}^{2}}\right)}{4\pi \;h\;\ln\left(\frac{R_{o}}{R_{i}}\right)}G\;\mathrm{and}\;\mu =\frac{\left(\frac{1}{R_{i}^{2}}-\frac{1}{R_{o}^{2}}\right)}{8\pi^{2}h}H$$

However, an alternative calculation method for the rotational rheometer, specifically designed for devices like the RheoCAD, has been developed independently from the initial approach outlined in Equation (2). This alternative method offers a significant advantage by resolving a limitation inherent in the first method, namely, its inability to accurately determine the shear rate/shear stress relationship. The detailed formulations underpinning this advanced calculation approach are presented in Equations (3) and (4) [51,52]. 


(3) $$\dot{\gamma }=\frac{R_{o}^{2}+R_{i}^{2}}{R_{o}^{2}-R_{i}^{2}}\times 2\pi \;N$$

(4) $$\tau =\frac{R_{o}^{2}+{\;R}_{i}^{2}}{4\;\pi \;h\;R_{o}^{2}R_{i}^{2}}\times T$$

This study will employ both calculation methods to provide a comprehensive overview of different approaches applied to the same materials. The objective is to compare and evaluate their effectiveness, ultimately identifying the most suitable method for calculating the characteristic coefficients of mortar. This comparative analysis will offer valuable insights into the strengths and limitations of each method, contributing to the optimization of rheological measurements and the selection of the best approach for accurately characterizing Bingham fluids.

**Figure 4:**
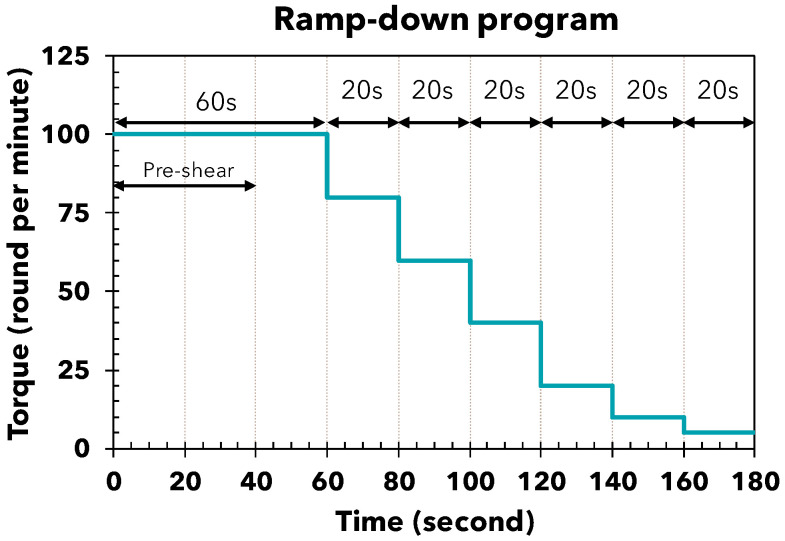
The ramp-down program for the RheoCAD.

To perform these rheological measurements, a dedicated rheometer program is employed that uses a ramp-down stress protocol [10] specifically designed for the blade rheometer. This protocol begins with a pre-stress phase to ensure material homogeneity, followed by a gradual decrease in shear stress every 20 s. The process continues until the shear stress reaches zero rounds per minute (Figure 4). 

## 3. Results 

### 3.1. Flow Table Test

**Figure 5:**
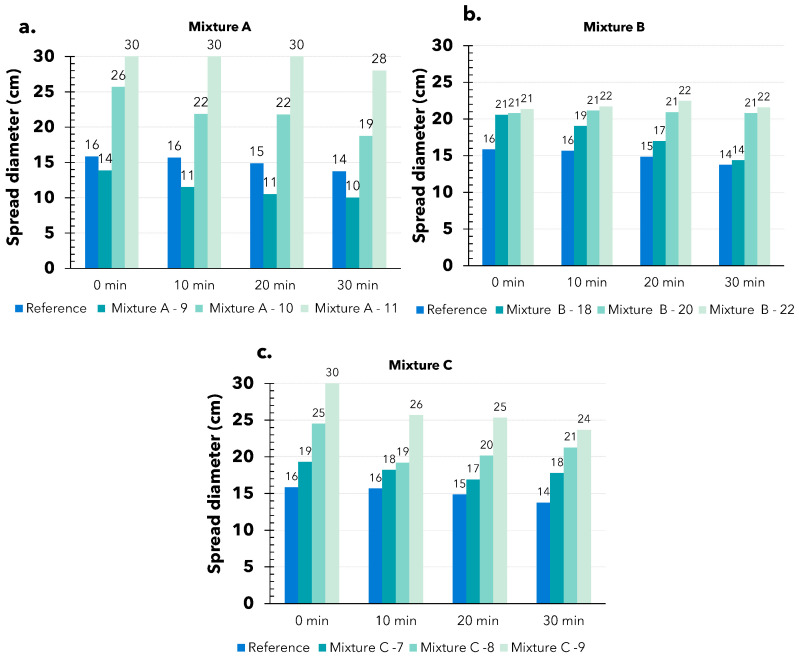
Results of the flow table test of difference mixtures: (**a**) A mixture with 9, 10, and 11% water content; (**b**) B mixture with 18, 20, and 22% water content; (**c**) C mixture with 7, 8, and 9% water content.

The reference mortar typically exhibits a much lower spread diameter compared to the 3D-printed mortar (Figure 5). This difference is expected knowing that the 3D printing process necessitates a material that can be transported smoothly through the printing pipe. Generally, 3D-printed mortar achieves a spread diameter of approximately 20–25 cm, which may represent the optimal value for such materials. For mixture A, the spread diameter changes significantly with variations in water content. With 9% water, the spread diameter is around 10–14 cm. However, with just a 1% or 2% increase in water, it rises to approximately 22–26 cm and 30 cm, respectively (Figure 5a). Meanwhile, mixture B exhibits the opposite behavior. Most of the oscillations fall within the range of 17 to 22 cm. As shown in Figure 5b, these values clearly indicate that mixture B maintains very stable behavior over time, even when the water content changes. For mixture C, the changes in these values are not as sensitive as in mixture A, but are also not as stable as in mixture B. In general, after 30 min of mixing, the mortar’s behavior remains stable, with flowability changing by only 1–6 cm (Figure 5c). Additionally, when more water is added, the flowability increases accordingly. 

The suitability of this test for assessing the behavior of 3D-printed materials seems to be interesting. The results provide insight into the material’s behavior over time across all mixes and highlight the differences between conventional mortar and 3D-printed mortar. The test also determined the ideal spread diameter for the 3D-printing process, matching the value of a commercial product with its original water content (18–20 cm). This finding is crucial and beneficial for practical applications. With this range of flow values, the printability of the material on the construction site can easily be evaluated with just a few simple steps. 

### 3.2. V-Funnel Test

**Figure 6:**
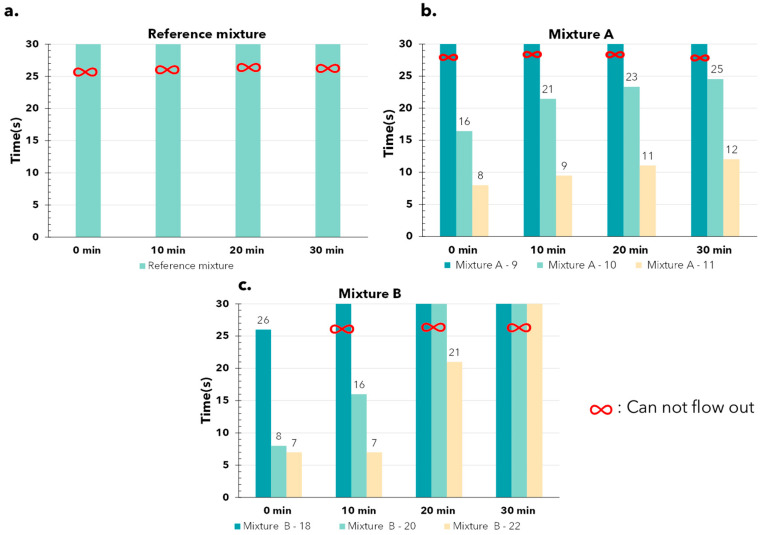
The results of the V–funnel test: (**a**) reference mixture; (**b**) A mixture with 9, 10, and 11% water content; (**c**) B mixture with 18, 20, and 22% water content.

The second test for 3D-printed mortar consistency is the V-funnel test. This test was performed at 0, 10, 20, and 30 min after mixing to evaluate the changes in the mortar over time (Figure 6). 

The reference mix could not be tested due to not flowing out of the funnel (Figure 6a), which is understandable given its poor flowability compared to the other mixes, as already observed in Figure 5. Mixture A with 9% water could not flow out of the funnel like the reference mixture. In contrast, Mixture A with 10% water took about 20 s to flow out, while the mixture with 11% water flowed out in an average of only 10 s (Figure 6b). These results clearly demonstrate the difference in flowability of the material at varying water contents. Unfortunately, with mixture B, this difference is not very evident because the material cannot flow out of the funnel (Figure 6c). This does not provide us with enough data to assess the material’s behavior. Based on just two mixes, it can be concluded that this test is unsuitable for 3D-printed concrete (mortar), as only mixtures with good flowability can pass through the funnel. In contrast, the properties of 3D-printed concrete are quite different; it often exhibits high viscosity after passing through the print nozzle, and the setting process of some materials can begin very quickly. For instance, mixture B, which contained 18% water, clogged the funnel after just 10 min, as shown in Figure 6c. Due to this incompatibility, mix C was not tested to prevent wasting time and materials.

In general, the V-funnel test revealed differences in material behavior among well-flowing mixes. However, it is evident that the behavior of 3D-printed mortar is significantly different and does not align with the results of the V-funnel test.

### 3.3. Fall Cone Test

**Figure 7:**
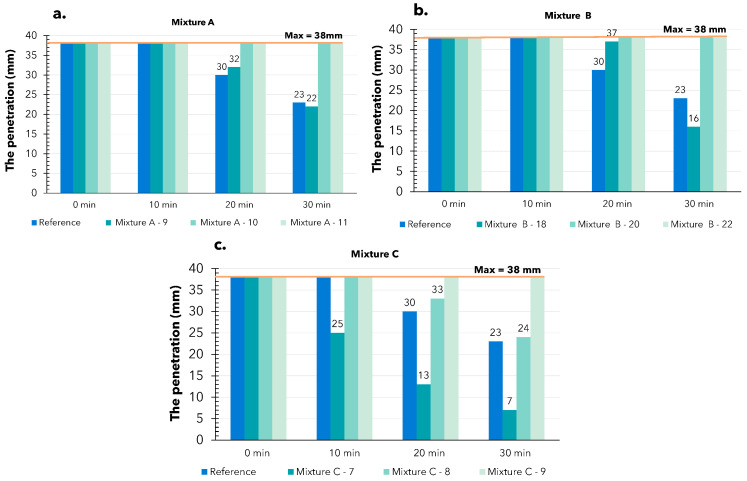
The results of fall cone test: (**a**) A mixture with 9, 10, and 11% water content; (**b**) B mixture with 18, 20, and 22% water content; (**c**) C mixture with 7, 8, and 9% water content.

With the fall cone test, the penetration of the cone into the material is measured for determining the printability of the material: The deeper the cone penetrates the material, the better the flowability of the material and vice versa. The maximum penetration depth of the cone is 38 mm. The results are shown in Figure 7. The test was, again, performed at 0, 10, 20, and 30 min after mixing to evaluate the changes in mortar over time.

The penetration of the cone tip with the standard mix was very high, reaching a maximum of 38 mm at 0 and 10 min, and then measuring 30 mm and 23 mm at 20 and 30 min, respectively. The penetration consistently reached about 70% of the maximum, indicating that the material exhibited minimal resistance to the cone drop. The same behavior was observed with the other mixes.

For mixture A (Figure 7a), the maximum penetration occurred within 10 min, then decreased to 32 mm and 23 mm at 20 and 30 min, respectively, with a water content of 9%. The other mixture always reached the maximum penetration. 

Mixture B (Figure 7b) consistently showed a maximum penetration of 38 mm, except at the 30 min, where, with the mix containing 18% water, this value decreased to 16 mm.

In contrast, mixture C demonstrated significantly greater resistance compared to the previous two mixes. Maximum penetration was only observed at a high water content in the mix at 0 min post-mixing. The mix with 7% water content showed good resistance to the cone, with penetration measuring only 25 mm, 13 mm, and 7 mm at 10, 20, and 30 min, respectively. At a higher water content, penetration remained very high (Figure 7c).

The results obtained from the experiment indicate that the weight of the cone tip was relatively too large compared to the materials’ resistance capacity. As a result, it consistently sunk to the bottom of the sample, making it difficult to observe changes in the material behavior at the initial time points. Unfortunately, for these materials, the fall cone test did not provide the necessary factors to evaluate the rheological behavior of printed mortar, making it an unsuitable test.

### 3.4. Pistol Test

Three-dimensional printing technology is based on additive manufacturing, and one of the best ways to evaluate the printability of 3D-printed samples seems to be the pistol test. Although this test is still very new and lacks standardized procedures, some research has identified key criteria for assessment [34,35]:

- ***Extrudability***: The ability to produce a continuous printed filament.
- ***Pumpability***: The ability to be transported efficiently within the pumping system.
- ***Buildability***: The ability to overlay multiple layers without deformation.

This test was performed at 0, 10, 20, and 30 min after mixing to evaluate the modifications of the mortar over time. 

**Figure 8:**
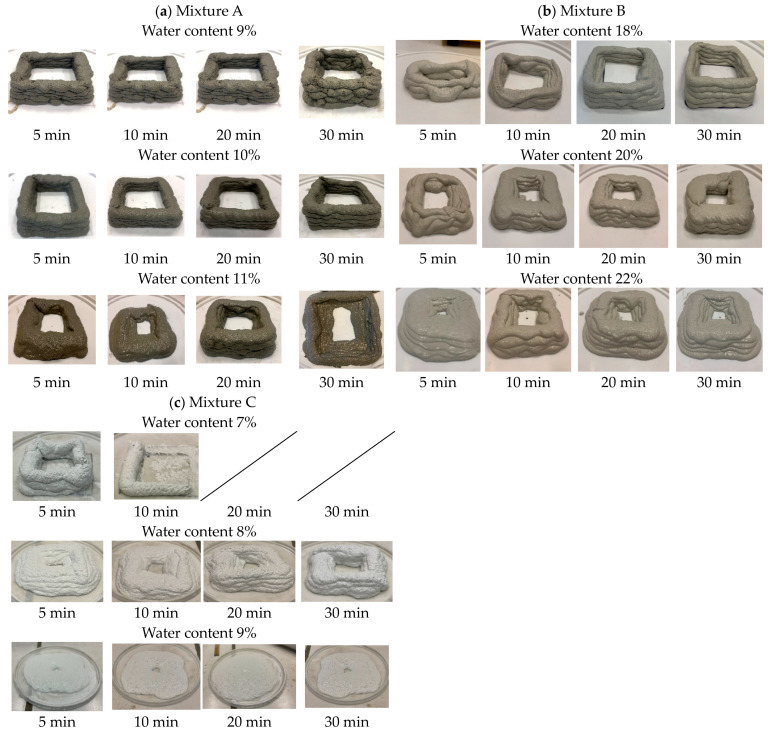
The pistol test results: (**a**) Mixture A with 9, 10, 11% water content; (**b**) mixture B with 18, 20, 22% water content; (**c**) mixture C with 7, 8, 9% water content.

For mixture A, 10% exhibited superior printability compared to the other tested formulations (Figure 8a). This material maintained remarkable extrudability, with its properties remaining unchanged even 30 min post-mixing, like those at the 5 min interval. Additionally, it demonstrated excellent buildability, effectively supporting the stacking of more than five layers without any issues. However, altering the water content caused the material to become either too dry or too wet, depending on whether the water was decreased or increased. This indicates that the mixture is sensitive to changes in water content, in agreement with the observations made during the flow table test.

With the commercial product, the B mixture (Figure 8b), optimal print performance was observed with 18% water content, specifically at 20 and 30 min post-mixing. Initially, extrudability and pumpability were achieved, but the mixture was too liquid for effective buildability. With 20% water content, the material’s initial behaviors were unsuitable for printing. However, as time progressed, the printability improved, allowing for the creation of smooth, continuous lines, and good buildability. At a 22% water content, the material was excessively liquid, compromising the stacking capability as the printed layers tended to collapse and merge.

The final mix deemed suitable for this experiment was the C mixture. Formulations with 7% (could not print after 20 min—Figure 8c) and 9% water content were clearly inadequate for 3D printing, as they failed to meet the necessary criteria for successful extrusion and buildability. A water content of 8% proved to be more effective. This mix exhibited good extrusion ability, ensuring that the material could be dispensed consistently during the printing process. Despite this, its buildability fell short when compared to the stability and performance of the B mixture. The printed lines produced with the 8% water content mix were still relatively large and prone to collapsing, making it difficult to print multiple layers reliably (Figure 8c).

### 3.5. Rheometer-RheoCAD

**Figure 9:**
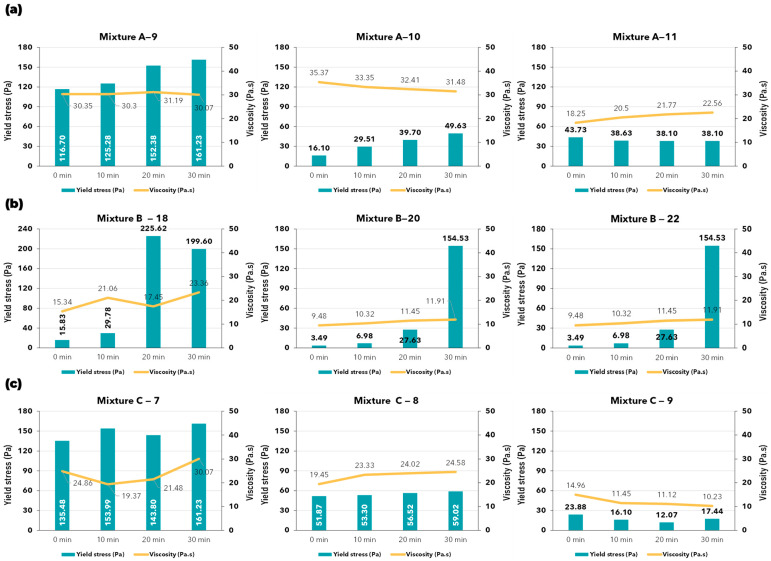
Yield stress and viscosity values: (**a**) A mixture with 9, 10, and 11% water content; (**b**) B mixture with 18, 20, and 22% water content; (**c**) C mixture with 7, 8, and 9% water content.

**Figure 10:**
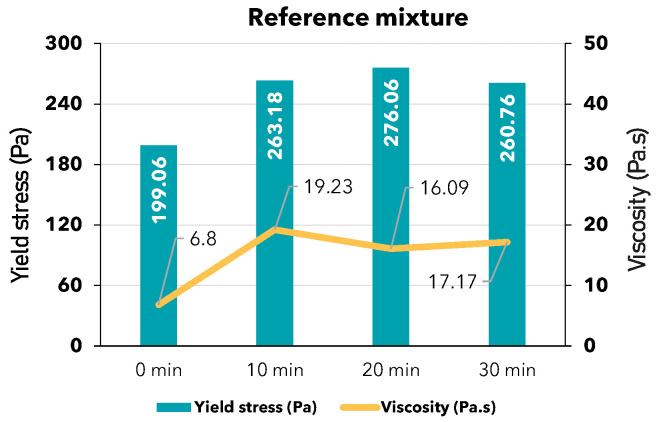
Yield stress and viscosity of reference mixture.

RheoCAD is an essential tool for precisely measuring the viscosity and yield stress of mortar. These measurements are critical in rheological studies, offering valuable insights into the material’s behavior. Figure 9 shows the values of the yield stress and the viscosity determined over time for all 3D-printed mixtures and the different water quantities tested according to Equation (2). Figure 10 illustrates the same value for the reference mixture. 

The reference mixture (Figure 10) exhibited a high yield stress value. According to theory [29,53,54,55,56,57,58], materials with high yield stress require a high shear stress to initiate movement, making transportation challenging. Therefore, a high yield stress material like the reference mixture is unsuitable for 3D printing.

In contrast, the commercial products mixed with the original water content have an initial yield stress value lower than that of the reference mixture (Figure 9). Regarding viscosity, these mixtures maintain a very stable value over time, approximately between 15 Pa·s and 30 Pa·s. This behavior has been verified as suitable for 3D-printed mortar in the pistol test. Comparing the reference mixture to the commercial products highlights the differences between a standard product and those specifically designed for this technology.

When comparing printable mixes, attention should be paid to their stability over time. The longer the properties remain consistent after mixing, the better the construction process. In this study, the 3D-printed mixes can maintain both yield stress and viscosity for up to 30 min after mixing for the A and C mixtures (Figure 9a,c—mixtures A and C), and up to 20 min for the B mixture—20% and 22% (Figure 9b)—which are suitable durations for practical printing processes. 

Identifying an optimal value for the 3D-printing process is very challenging. The behavior of the material can be affected by various factors, such as the diameter of the pipe, the pump pressure, the composition design, the speed of the nozzle, etc. In general, a mixture for 3D printing construction should have low yield stress [59,60] for easier transport (pumpability), but it also needs appropriate viscosity and yield stress [26,35,61,62] to meet 3D printing requirements after printing (buildability). Therefore, in addition to using a rheometer, it is crucial to conduct tests to evaluate these requirements, such as a pistol test or running a trial on an actual 3D printer.

### 3.6. Correlation of RheoCAD Rheological Values with Classical Test

The flow table test, V-funnel test, and pistol test were the three basic tests conducted. It is evident that the V-funnel test and the fall cone test are not suitable for this study. The remaining two tests, the flow table test and pistol test, are appropriate and can be combined with rheometer results.

However, it is currently not reasonable to correlate the results of the pistol test with rheological properties. After removal of the extruded mortar bead, the behavior of concrete is in physical transition to a semi-solid state: the solidification process begins and parameters such as yield stress and viscosity lose significancy for describing the extruded mortar’s behavior. Therefore, only the results of the flow table test are used for comparison with yield stress and viscosity measured with the rheometer.

**Figure 11:**
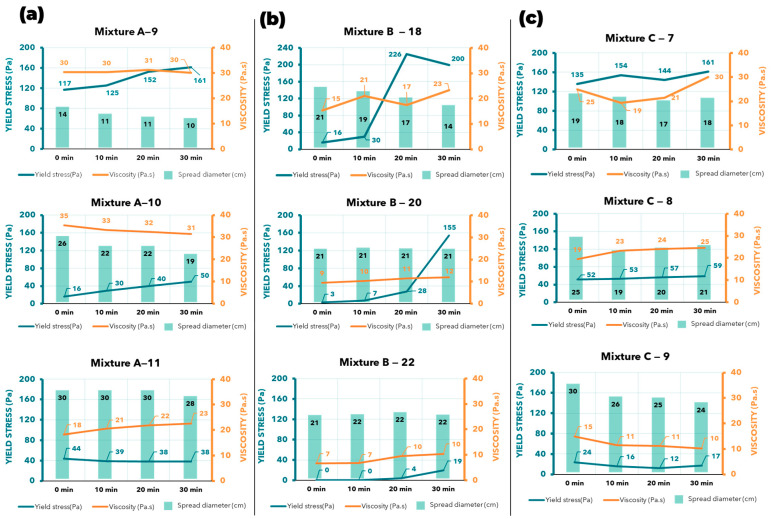
The combination of rheometer (RheoCAD) results and spread diameter from the flow table test: (**a**) A mixture with 9, 10, and 11% water content; (**b**) B mixture with 18, 20, and 22% water content; (**c**) C mixture with 7, 8, and 9% water content.

The relationship between yield stress and spread diameter is highly unpredictable. For example, mixture A-9 (Figure 11a) showed a spread diameter of about 10–14 cm, corresponding to a yield stress of 117–161 Pa. However, mixture C-7 (Figure 11c), with a similar yield stress range, had a much larger spread diameter of 17–19 cm, representing a significant difference in material behavior. Even within the same mix, this inconsistency is evident. For instance, mixture B-18 (Figure 11b) showed a linear decrease in spread diameter over time, from 21 to 14 cm, yet the yield stress fluctuated unpredictably, jumping from 30 to 226 Pa between the 10 and 20 min marks. Although mixture A-10 (Figure 11a) followed a linear rule—where a smaller spread diameter corresponded to a higher yield stress—this relationship does not hold consistently across other mixes.

The viscosity shows better correlation with the spread diameter. For example, the smaller the spread diameter, the higher the viscosity, and vice versa. In mixture A-9 (Figure 11a), a spread diameter of 11–14 cm corresponded to a viscosity of about 30 Pa·s, and both values remained stable over time. In contrast, mixture A-11 (Figure 11a) showed an increased spread of 30 cm across all tests, with a corresponding decrease in viscosity to 18–22 Pa·s. However, there are exceptions, such as in mixture B-18 (Figure 11b), where the viscosity fluctuated, but the spread diameter steadily decreased from 21 to 14 cm. A similar pattern was observed in mixture C-7 (Figure 11c).

Overall, for each spread diameter, we can reasonably predict the corresponding viscosity, even though the prediction may not be 100% accurate. This relationship is still useful for determining the printability of the material. For example, in mixture C-9 (Figure 11c), a spread diameter of 25 cm corresponded to a viscosity of 10–11 Pa·s, while 30 cm corresponded to a viscosity of around 15 Pa·s. It is important to note that this inference cannot be applied across different mixes. For instance, at the same viscosity of 10–11 Pa·s, mixture B-20 (Figure 11b) had a spread diameter of 21 cm, 4 cm smaller than that of mixture C-9. Each material has its own unique properties, and they do not share identical spread diameter values.

## 4. Discussion

### 4.1. The Consistency Test for 3D-Printing Mortar and Correlation with Rheological Properties

The results from the above experiments demonstrate that the flow test and pistol test are effective for assessing the quality of 3D-printed mortar. The flow table test successfully monitored material behavior over different time intervals, while the pistol test was crucial, as it assessed three critical properties of the material: pumpability, extrudability, and buildability. Although the V-funnel test provided some useful insights into the material’s flowability, it proved unsuitable at higher yield stress levels, where the material could not exit the funnel. Similarly, although previous studies [63] suggest that the fall cone test is suitable for 3D-printed mortar, it was ineffective in this study. The cone’s penetration was too deep relative to the material’s resistance, making it difficult to evaluate the material’s printability at the initial stage.

It is important to note that the unsuitability of the V-funnel test and fall cone test in this study does not imply that these methods are entirely inappropriate for 3D-printed concrete. The suitability of these tests depends on the specific properties of each material and the design of the test apparatus. For example, in the study by Pham et al. [12], the flowability of the 3D-printed material they investigated was notably better than in other studies. Similarly, in the research by Rehman et al. [63], the fall cone test proved to be highly appropriate due to their material’s relatively high yield stress, around 1–2 kPa. Additionally, their study employed advanced equipment, enabling precise measurements and the direct measurement of parameters such as the shear yield stress, force, cone penetration, etc.

In combining rheological factors with experimental tests, only the flow table test is suitable for such an integration. The spread diameter values are closely related to the material’s viscosity, allowing flowability to serve as a reliable indicator for predicting material properties. The relationship between the pistol test and rheological parameters is more complex and cannot be clearly demonstrated in this study. First, a more precise printing system, as described in [64], is required to establish a meaningful relationship with other tests. Additionally, many studies [65,66,67] have shown that the behavior of printed mortar is more dependent on solid material parameters, such as Young’s modulus and Poisson’s ratio. To accurately assess the material’s buildability, solid mechanics laws have been found to provide more accurate evaluations than applying liquid parameters. Furthermore, pumpability and printability are subjective parameters that are challenging to quantify using the equipment employed in this study. To properly assess these parameters, modeling studies on the stability of the printed elements and the material’s behavior within the pump are essential. 

### 4.2. Comparison of Two Mathematical Models for Determining Yield Stress and Viscosity

In this study, two methods are utilized to calculate yield stress and viscosity based on the results obtained from the RheoCAD device. The results presented in the previous section are derived from the first method. In this section, a comparison of the results from both methods will be conducted.

**Figure 12:**
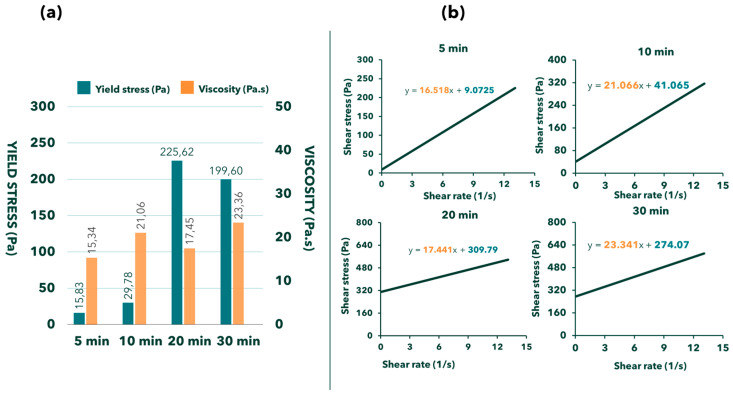
The comparison between the first method (**a**) and the second method (**b**) or calculating the rheological characteristics of 371—SAB—25–18% water content.

The primary mathematical model for determining rheological parameters utilizes Equation (2). This method is beneficial due to its straightforwardness and its capacity to directly compute the two most crucial parameters: yield stress and viscosity (Figure 12b). However, a significant drawback is that the results of this calculation method do not incorporate the Bingham model. As a result, it provides incomplete insights into the material’s behavior or the corresponding equation needed to fit the Bingham model, which may lead to difficulties in analyzing the research results. Therefore, a secondary mathematical model was employed for further using Equations (3) and (4). This alternative calculation method allows for the initial computation of the shear rate and shear stress (Figure 12a). Subsequently, all values can be plotted graphically, facilitating the determination of yield stress and viscosity. This method clearly illustrates the Bingham behavior, enables a comprehensive understanding of the model, and simplifies the connection with theoretical concepts. 

Apart from the obvious benefits of clearly representing the theoretical material behavior, there is another notable difference in the values of yield stress and viscosity when comparing the second calculation method with the first one. Table 3 highlights these differences with the use of Equation (5):

(5) $$\%\;\mathrm{Difference}=\frac{\mathrm{value}\;\mathrm{of}\;\mathrm{the}\;\mathrm{second}\;\mathrm{method}\text{-}\mathrm{value}\;\mathrm{of}\;\mathrm{the}\;\mathrm{first}\;\mathrm{method}}{\mathrm{value}\;\mathrm{of}\;\mathrm{the}\;\mathrm{first}\;\mathrm{method}}\times 100\%$$

As shown in Table 3, the differences between the mixes are relatively small in terms of viscosity, but quite significant in terms of yield stress. However, there are exceptions; for instance, mix C-8 exhibits opposite behavior compared to the other cases, with a substantial difference in viscosity between the two methods (25%) but a minimal difference in yield stress (less than 3%) (Table 3). Such cases, though rare, can be considered outliers and may be disregarded. Therefore, the primary distinction between the mixes is the variation in yield stress. Determining which method is more accurate and should be permanently adopted for RheoCAD is challenging. Further studies with careful measurements of these quantities are necessary to address this question.

## 5. Conclusions

The following conclusions may be drawn from the present investigations concerning the suitability of tests for evaluating the capacity of mortars to be used for 3D printing:

1. The flow table test is the most efficient empirical method for evaluating the consistency of 3D-printed mortar and its extrudability. It successfully demonstrates the mortar rheological property’s evolution over time and is easy to perform on construction sites. In contrast, the V-funnel and fall cone tests do not offer comparable insights.
2. The pistol test is essential for 3D-printing construction, as it helps in the determination of the extrudability and buildability properties and predicts potential pumping issues in real printers. However, it is difficult to correlate to rheological parameters such as viscosity and shear stress.
3. The RheoCAD rheometer is an accurate device for measuring rheological parameters, specifically yield stress and viscosity. Understanding these values is crucial for analyzing mortar behavior in the pumping systems and addressing pipe and printer issues. Specific values of viscosity and shear stresses are needed for proper mortar transportation in the pipe.
4. The correlation between a rheometer (RheoCAD) and a simple test (flow table test) presents a promising research avenue. This combination enhances the understanding of flow table test results in terms of theory based on yield stress and viscosity. The study indicates how the flow table test is more closely related to viscosity than yield stress, as shown by the correlation between spread diameter and viscosity over time.
5. There are two methods for calculating results from the rheometer. Generally, these methods provide similar viscosity values, but a huge difference in yield stress values (30–40% difference). The method using Equations (3) and (4) is more intuitive, as it illustrates mortar behavior and aligns with the mechanical fluid theory for Bingham models. Conversely, the method using Equation (2) is more straightforward for calculating yield stress and viscosity.

Further investigations are needed for extending the generalization of conclusions to specific mortar and modeling the behavior of fresh mortar inside the pump system.

## Figures and Tables

**Table 1:** Detailed characteristics of commercial products.

	Product Type	A Mixture	B Mixture	C Mixture
Properties				
Binder		Portland Cement	Portland Cement	Calcium aluminate & reactive alumina
Maximum particle size (mm)		1.60	1	5
Density (g/cm^3^)		2.20	2.14	2.90
Initial setting time (h)		8.50	0.75	/
Final setting time (h)		10	1.08	/
Compressive strength (MPa—28 days)		60	50	40

**Table 2:** References of mixtures and water content.

Mixture Number	Mixture ID	Water Content (wt.%)
1	Reference	11.13%
2	A—9	9%
3	A—10	10%
4	A—11	11%
5	B—18	18%
6	B—20	20%
7	B—22	22%
8	C—7	7%
9	C—8	8%
10	C—9	9%

**Table 3:** The difference between the first method and the second method in all the mixtures of the research.

		First Method		Second Method		Difference (%)	
Mixture ID	Time (min)	Yield Stress (Pa)	Viscosity (Pa·s)	Yield Stress (Pa)	Viscosity (Pa·s)	Yield Stress (Pa)	Viscosity (Pa·s)
**Reference**	0	199.06	6.8	273.27	6.79	37.28%	−0.2%
	10	263.18	19.23	361.47	19.21	37.35%	−0.1%
	20	276.06	16.09	378.97	16.08	37.28%	−0.1%
	30	260.76	17.17	358.16	17.15	37.35%	−0.1%
**A—9**	0	116.7	30.35	160.42	30.37	37.46%	0.1%
	10	125.28	30.3	171.93	30.28	37.24%	−0.1%
	20	152.38	31.19	209.24	31.22	37.31%	0.1%
	30	161.23	30.07	236.20	28.19	46.50%	−6.3%
**A—10**	0	16.1	35.37	22.17	35.37	37.67%	0.0%
	10	29.51	33.35	40.53	33.35	37.35%	0.0%
	20	39.7	32.41	54.44	32.43	37.13%	0.0%
	30	49.63	31.48	68.26	31.47	37.53%	0.0%
**A—11**	0	43.73	18.25	60.09	18.24	37.42%	−0.1%
	10	38.63	20.5	53.07	20.50	37.38%	0.0%
	20	38.1	21.77	52.43	21.77	37.61%	0.0%
	30	38.1	22.56	52.29	22.55	37.24%	0.0%
**B—18**	0	15.83	15.34	9.07	16.52	−42.69%	7.7%
	10	29.78	21.06	41.07	21.07	37.89%	0.0%
	20	225.62	17.45	309.79	17.44	37.31%	−0.1%
	30	199.6	23.36	274.07	23.34	37.31%	−0.1%
**B—20**	0	3.49	9.48	4.83	9.46	38.50%	−0.2%
	10	6.98	10.32	9.62	10.33	37.77%	0.1%
	20	27.63	11.45	38.18	11.41	38.18%	−0.3%
	30	154.53	11.91	217.49	11.46	40.74%	−3.8%
**B—22**	0	0	6.71	0.00	6.72	0.00%	0.1%
	10	0	6.75	0.00	6.77	0.00%	0.3%
	20	3.76	9.52	5.12	9.52	36.15%	0.0%
	30	19.32	10.32	26.63	10.34	37.84%	0.2%
**C—7**	0	135.48	24.86	185.95	24.88	37.25%	0.1%
	10	153.99	19.37	211.45	19.39	37.31%	0.1%
	20	143.8	21.48	197.61	21.50	37.42%	0.1%
	30	161.23	30.07	203.92	22.61	26.48%	−24.8%
**C—8**	0	51.87	19.45	53.37	14.58	2.89%	−25.1%
	10	53.3	23.33	54.82	17.47	2.85%	−25.1%
	20	56.52	24.02	58.11	18.02	2.82%	−25.0%
	30	59.02	24.58	60.61	18.46	2.69%	−24.9%
**C—9**	0	23.88	14.96	32.79	14.98	37.33%	0.1%
	10	16.1	11.45	21.95	11.43	36.32%	−0.2%
	20	12.07	11.12	16.52	11.10	36.83%	−0.2%
	30	17.44	10.23	23.95	10.20	37.31%	−0.3%

## Data Availability

The original contributions presented in the study are included in the article, further inquiries can be directed to the corresponding author.
